# Treatment of Barber’s Itch

**Published:** 1881-04

**Authors:** 


TREATMENT OF BARBER'S ITCH.—Brame recommends the following treatment: Shave off the hairs or cut them very short; then apply once or twice a week an ointment composed of: ℞.—Prepared chalk, 10 parts; coal tar, 1 to 4 parts; glycerine, 5 parts; simple cerate, 40 parts

